# Supplementary material for: Poincaré maps for analyzing complex hierarchies in single-cell data
Source: Nat Commun. 2020 Jun 11;11:2966. doi: 10.1038/s41467-020-16822-4 (PMC7290024; doi:10.1038/s41467-020-16822-4)
Supplement: Supplementary file 1 — Supplementary Information [file 41467_2020_16822_MOESM1_ESM.pdf]

Supplementary Information

Poincaré Maps for Analyzing Complex Hierarchies in Single-Cell Data

Klimovskaia et al.

Supplementary Note 1: Poincaré maps for learning hierarchical representations

Hyperbolic spaces are a Riemannian manifolds whose structure is well-suited to represent hierarchical and tree-like relationships. For our work, this combines two important advantages: First, the metric structure of hyperbolic spaces allows us to capture continuous hierarchical relationships and interpolate between points. Second – and in contrast to other metric spaces – hierarchies can already be represented in two-dimensional hyperbolic space with small distortion [1, 2, 3, 4].

Poincaré disk model

There exist multiple, equivalent models of hyperbolic spaces, such as the Beltrami-Klein, the Lorentz, and the Poincaré half-plane model. In this work, we base our approach on the Poincaré disk model, as it is best suited for visual analysis. The Poincaré disk is defined as follows: let $\mathcal{P} = \{\boldsymbol{x} \in \mathbb{R}^2 \mid \|\boldsymbol{x}\| < 1\}$ be the *open* unit disk, where $\| \cdot \|$ denotes the Euclidean norm. The Poincaré disk corresponds then to the Riemannian manifold $(\mathcal{P}, g_{\boldsymbol{x}})$, i.e., the open unit disk equipped with the Riemannian metric tensor

$$g_{\boldsymbol{x}} = \left(\frac{2}{1 - \|\boldsymbol{x}\|^2} \right)^2 g^E,$$

where $\boldsymbol{x} \in \mathcal{P}$ and g^E denotes the Euclidean metric tensor. Furthermore, the distance between points $\boldsymbol{u}, \boldsymbol{v} \in \mathcal{P}$ is given as

$$d(\boldsymbol{u}, \boldsymbol{v}) = \operatorname{acosh}\left(1 + 2 \frac{\|\boldsymbol{u} - \boldsymbol{v}\|^2}{(1 - \|\boldsymbol{u}\|^2)(1 - \|\boldsymbol{v}\|^2)} \right). \tag{1}$$

The boundary of the disk is denoted by $\partial\mathcal{B}$ and is not part of the manifold, but represents infinitely distant points. Geodesics in $\mathcal{P}$ are then arcs orthogonal to $\partial\mathcal{B}$ (as well as all diameters). See **??** for an illustration.

It can be seen from Equation (1) that the Euclidean distance of two points in the Poincaré disk is amplified with respect to their distance to the origin of the disk. This locality property of the Poincaré distance is key for continuous embeddings of hierarchies. For instance, by placing the root node of a tree at the origin of $\mathcal{B}^d$ it would have a relatively small distance to all other nodes as its Euclidean norm is zero. On the other hand, leaf nodes can be placed close to the boundary of the Poincaré disk, as the distance grows fast between points with a norm close to one. Furthermore, Equation (1) is symmetric and the hierarchical organization of the space is solely determined by the distance of points to the origin. Due to this property, Equation (1) is applicable in an unsupervised setting, where the hierarchical order of objects is not specified in advance. Importantly, this allows to learn embeddings that simultaneously capture the hierarchy of objects (through their norm) as well as their similarity (through their distance).

The Riemannian manifold structure of hyperbolic spaces enables the use Riemannian Stochastic Gradient Descent (RSGD) [5] to compute the embeddings. In RSGD, parameter updates are performed via

$$\boldsymbol{y}_{t+1} = \mathfrak{R}_{\boldsymbol{y}_t}(-\eta \operatorname{grad}(\mathcal{L}, \boldsymbol{y}_t))$$

where $\mathfrak{R}_{\boldsymbol{y}}$ denotes a retraction from the tangent space at $\boldsymbol{y}$ onto the manifold, $\operatorname{grad}(\mathcal{L}, \boldsymbol{y}_t)$ denotes the Riemannian gradient of the scalar function $\mathcal{L}$, and $\eta > 0$ denotes the learning rate. The embeddings can be learned directly in the Poincaré disk $\mathcal{P}$ or, alternatively, in the Lorentz model (which has advantageous properties for stochastic optimization). We refer to [2] and [4] for the detailed optimization procedure on both hyperbolic manifolds. When optimization is performed in the Lorentz model, we can map the learned embeddings into the Poincaré disk via the diffeomorphism $p : \mathcal{H} \to \mathcal{P}$, where:

$$p(x_0, x_1, \ldots, x_n) = \frac{(x_1, \ldots, x_n)}{x_0 + 1}$$

which preserves all geometric properties including isometry.

Supplementary Note 2: Benchmarks on datasets with known hierarchy

Visualization

We compare Poincaré maps to several methods frequently used for visualization: tSNE [6], UMAP [7], diffusion maps [8], graph abstractions (PAGA [9]), ForceAtlas2 [10] and Monocle 2 [11]. For all competing methods, we used a set of parameters in the range provided by the authors. For the visualization comparison, for each method we chose the best set of parameters in terms of quality metric described below.

While methods such as diffusion maps, PAGA and Monocle 2 can be used by a knowledgeable user to infer the correct structure form data with several post-processing iterations, here we would like to demonstrate how Poincaré maps extract meaningful insights from data without further post-processing. The ability to recover hidden hierarchies automatically and in one shot makes Poincaré maps an attractive tool for the analysis of branching processes and complex hierarchical structures.

Scale-independent quality criteria

To quantitatively compare the performance of different embedding approaches, we use a scale-independent quality criteria proposed by Lee et al. [12] The main idea is that a good dimensionality reduction approach will have a good preservation of local and global distances on the manifold, e.g. close neighbors should be placed close to each other while maintaining large distances between distant points. Below we provide a short summary of how to compute this metric. All the details can be found in the original paper of Lee et al.[12]

Let $\mathcal{X} = \{x_i\}_{i=1}^N$ be a high-dimensional dataset of N samples $\boldsymbol{x}_i \in \mathbb{R}^p$ (e.g., individual cells) with p features (e.g., gene expression measurements) and $\mathcal{Y} = \{y_i\}_{i=1}^N$ be a low-dimensional representation of this dataset in $m = 2$ dimensions. Let δ_{ij} denote the distance from x_i to x_j in the high-dimensional space and d_{ij} denote the distance from y_i to y_j in the low-dimensional space. Assume $\delta_{ij} = \delta_{ji}$ and $d_{ij} = d_{ji}$. The distances could be used to compute high ($R = \{\rho_{ij}\}_{1 \leq i,j \leq N}$) and low ($V = \{\nu_{ij}\}_{1 \leq i,j \leq N}$) dimensional ranks between the points:

$$\rho_{ij} = |k : \delta_{ik} < \delta_{ij} \text{ or } (\delta_{ik} = \delta_{ij} \text{ and } 1 \leq k < j \leq N)|, \tag{2}$$

$$\nu_{ij} = |k : d_{ik} < d_{ij} \text{ or } (d_{ik} = d_{ij} \text{ and } 1 \leq k < j \leq N)|, \tag{3}$$

where $|A|$ denotes the cardinality of a set. According to this definition, reflexive ranks are set to zero and non-reflexive ranks belong to $\{1, \ldots, N-1\}$.

A co-ranking matrix $\mathbf{Q} = \{q_{kl}\}_{1 \leq k,l \leq N-1}$ is defined as:

$$q_{ij} = |\{(i,j) : \rho_{ij} = k \text{ and } r_{ij} = l\}| \tag{4}$$

The co-ranking matrix contains all the necessary information about how ranks are preserved in a given low-dimensional representation. As was demonstrated by Lee et al. [12], the co-ranking matrix $\mathbf{Q}$ is straightforward to compute, and it could be used to compute Q_{NX} – scale-independent quality criteria for dimensionality reduction for a given value of $K = 1, \ldots, N-1$:

$$Q_{NX}(K) = \frac{1}{KN} \sum_{(k,l) \in \mathbb{UL}_{\mathbb{K}}} q_{kl}, \tag{5}$$

where $\mathbb{UL}_{\mathbb{K}} = \{1, \ldots, K\} \times \{1, \ldots, K\}$ is the upper left corner of co-ranking matrix. $Q_{NX}(K) \in [0, 1]$ assesses the overall quality of the embedding. Essentially, it measures the preservation of K-ary neighborhoods. A perfect embedding has $Q_{NX}(K) = 1$ for every $K = 1, \ldots, N-1$.

The left part of of the $Q_{NX}(K)$ curve reflects how local properties are preserved, and the right part corresponds to the preservation of global properties. To improve its readability, Lee et al.[12] propose to use two scalar quality criteria Q_{local} and Q_{global} focusing separately on low and hight dimensional qualities of the embedding:

$$Q_{local} = \frac{1}{K_{max}} \sum_{K=1}^{K_{max}} Q_{NX}(K), \tag{6}$$

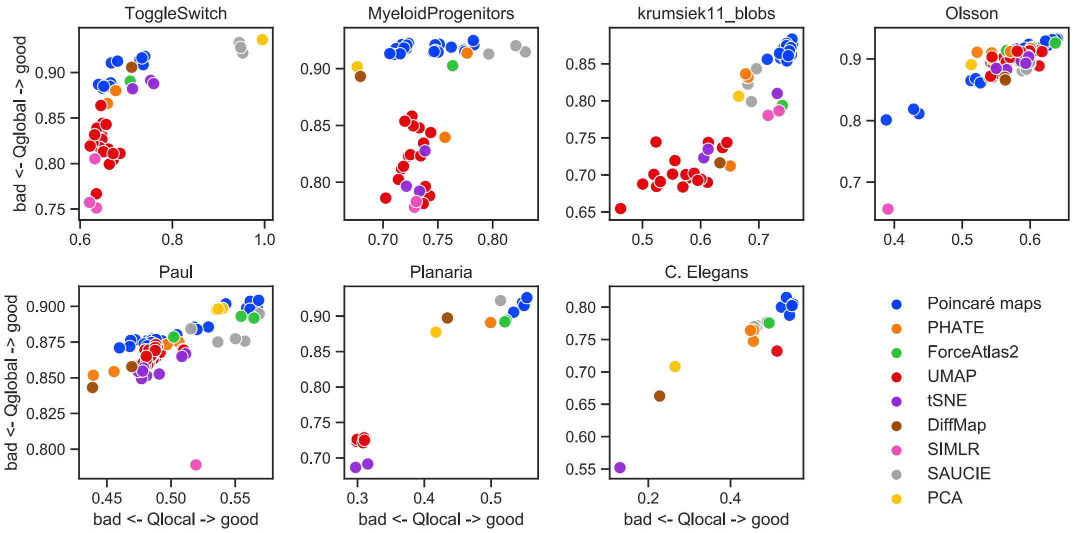

Supplementary Figure 1. Comparison of local and global quality metrics for various datasets. All embeddings were computed with 3 random seeds and several different hyperparameters. We fixed $k = 20$ for all the datasets for a fair comparison.

$$Q_{global} = \frac{1}{N - K_{max}} \sum_{K=K_{max}}^{N-1} Q_{NX}(K), \tag{7}$$

where K_{max} defines the split of the Q_{NX} curve and is automatically computed as:

$$K_{max} = \arg\max_{K} \left(Q_{NX}(K) - \frac{K}{N-1} \right) \tag{8}$$

The quantities of Q_{local} and Q_{global} range from 0 (bad) to 1 (good).

In this work, to estimate distances δ_{ij} in the high-dimensional space, we use geodesic distances estimated as the length of a shortest-path in a k-nearest neighbors graph. We fixed $k = 20$ for all the datasets, as there is no objective way to decide on a correct k, and visually results looked good for all the embeddings for this choice of k. For the distances δ_{ij} in the low-dimensional space we use euclidean distances for all the embeddings except Poincaré maps, for which we use hyperbolic distances. As all the embeddings involve an element of stochasticity in their output, we run every embedding three times with a different seed. We run all the embeddings with a different set of parameters in the range proposed by the authors of each method. For our comparisons, we used the scanpy implementation of PCA, UMAP, tSNE, and diffusion maps, as these are very effective implementations adapted for single-cell datasets. The scanpy package provides recommendations for the default set of parameters (demonstrated to work well on a wide range of single-cell datasets), so we tried all the recommended parameters. In particular, for UMAP we used $\gamma = 1.0, 2.0$, min_dist $= 0.1, 1.0, 0.5$, spread $= 0.1, 0.5, 1.0$. For tSNE, the scanpy implementation allows to vary perplexity, but since this parameter is linked to the k in k-nearest neighbors, we fix it for all the methods for a fair comparison. For ForceAtlas2, we used a PAGA initialization (with a resolution of 0.9, as recommended by the authors) as it was demonstrated to substantially improve the performance of the ForceAtlas2 method. Diffusion maps have only two parameters n_comp (number of dimensions) and k nearest neighbors, which are fixed between all the methods for a fair comparison. For SIMLR, we provided additional advantage by using information about the number of cell types: $c =$ number of cell types computed from annotated labels, cores.ratio $=$ 0. For PHATE, we used parameters recommended by the authors in their tutorial notebook: knn_dist="euclidean", gamma=0, t=12, decay=15. For SAUCIE, we used steps=1000.

Supplementary Figure 1 demonstrates the comparison of Q_{local} and Q_{global} for all the datasets described below.

Robustness of Poincaré maps to random seed and choice of hyper-parameters choice

We used the quality criteria described above and visual inspection to address the robustness of Poincaré maps to hyper-parameters choice and random seed. **Supplementary Figure 2 (a)** demonstrates that good values of σ vary for different datasets. However, the parameter γ has a less strong effect and rather controls how much the embedding will be scattered on the disk. We advise to set γ to 1.0 or 2.0 depending on the dataset size: larger datasets typically have better visualization with $\gamma = 2.0$ (**Supplementary Figure 2 (b-c)**). **Supplementary Figure 2 (a, d)** demonstrates that Poincaré maps are very robust to random seed and that it doesn't significantly affect neither quality nor visual interpretation.

Synthetic datasets

To demonstrate the performance of Poincaré maps we used several synthetic datasets available as Jupyter notebooks with Scanpy [13]: a simple toggle switch, myeloid progenitors and myeloid progenitors with Gaussian blobs. These datasets were previously used to demonstrate the performance of diffusion maps and graph abstractions, and constitute great examples of manifolds with a hierarchical structure of increasing levels of complexity. All models consist of Boolean equations, which were translated into ordinary differential equations and simulated with Scanpy as stochastic differential equations with Gaussian noise [14].

A simple toggle switch model [15, 16] is a process with two branches, which are defined by the expression of two markers. **Supplementary Figure 3** demonstrates that all competing methods produce rather correct results for this simple problem. However, Poincaré maps give a more clear separation of the intermediate states of terminal fates (inter1 vs inter2). In this example, only tSNE, diffusion maps, and Poincaré maps produce embeddings with meaningful pairwise distances.

A synthetic dataset for myeloid differentiation [17] represents cell differentiation progresses of a common myeloid progenitor state towards one of four different branches: erythrocyte, neutrophil, monocyte, and megakaryocyte. **Supplementary Figure 4** shows the provided embeddings for all methods. Poincaré maps produce an embedding which is visually similar to the other methods but has neither discontinuities nor overlaps in the trajectories since it preserves all the pairwise distances. Given the known root, the rotation of the Poincaré map (by means of translation) allows to easily read out the hierarchy. Diffusion maps produce embeddings consistent with one main branch, but more Euclidean dimensions would be necessary to separate the rest. Monocle 2 produces a tree layout consistent with the hierarchical structure of the data but is not able to reconstruct the temporal connection (trajectory) of the cell differentiation process.

The third dataset shows the stability of Poincaré maps with respect to the existence of clusters not related to the main cell development process. To this end we use the synthetic dataset of myeloid differentiation with two Gaussian blobs, added as proposed by Wolf et al. [9] (**Supplementary Figure 5**). None of the benchmark methods except ForceAtlas2 is able to capture the hierarchy.

Mouse myelopoesis dataset (single-cell RNA seq)

To demonstrate the performance of Poincaré maps on single-cell RNA seq data, we used the mouse myelopoesis dataset (wild type only) from Olsson et al. [18]. The data was downloaded and preprocessed according to the pipeline from Qiu et al. [11]. The processed dataset contained 532 features for 382 cells. Nine cell types were annotated corresponding to the original study: HSCP-1 (hematopoietic stem cell progenitor), HSCP-2, megakaryocytic, erythrocytic, Multi-Lin* (multi-lineage primed), MDP (monocyte-dendritic cell precursor), monocytic, granulocytic and myelocyte (myelocytes and metamyelocytes). In order to obtain the best results for Monocle 2, we used the analysis pipeline provided by the authors (`https://github.com/cole-trapnell-lab/monocle2-rge-paper`). As the reference hierarchy, we used the canonical hematopoetic cell lineage tree [19] (**Supplementary Figure 6 (a)**).

Poincaré maps, after rotation (**Supplementary Figure 6 (b)**), reveal the known hierarchy and suggest that part of HSPC-2 cluster actually corresponds to the megakaryocyte/erythrocyte progenitor (MEP), and that the cluster named Multi-Lin corresponds to the granylocyte/monocyte progenitor (GMP). Also according to Poincaré maps, the cluster annotated as myelocyte does not belong to the hierarchy, or constitutes a mature state of granulocytes. However, the validation of these hypotheses requires a detailed differential expression analysis.

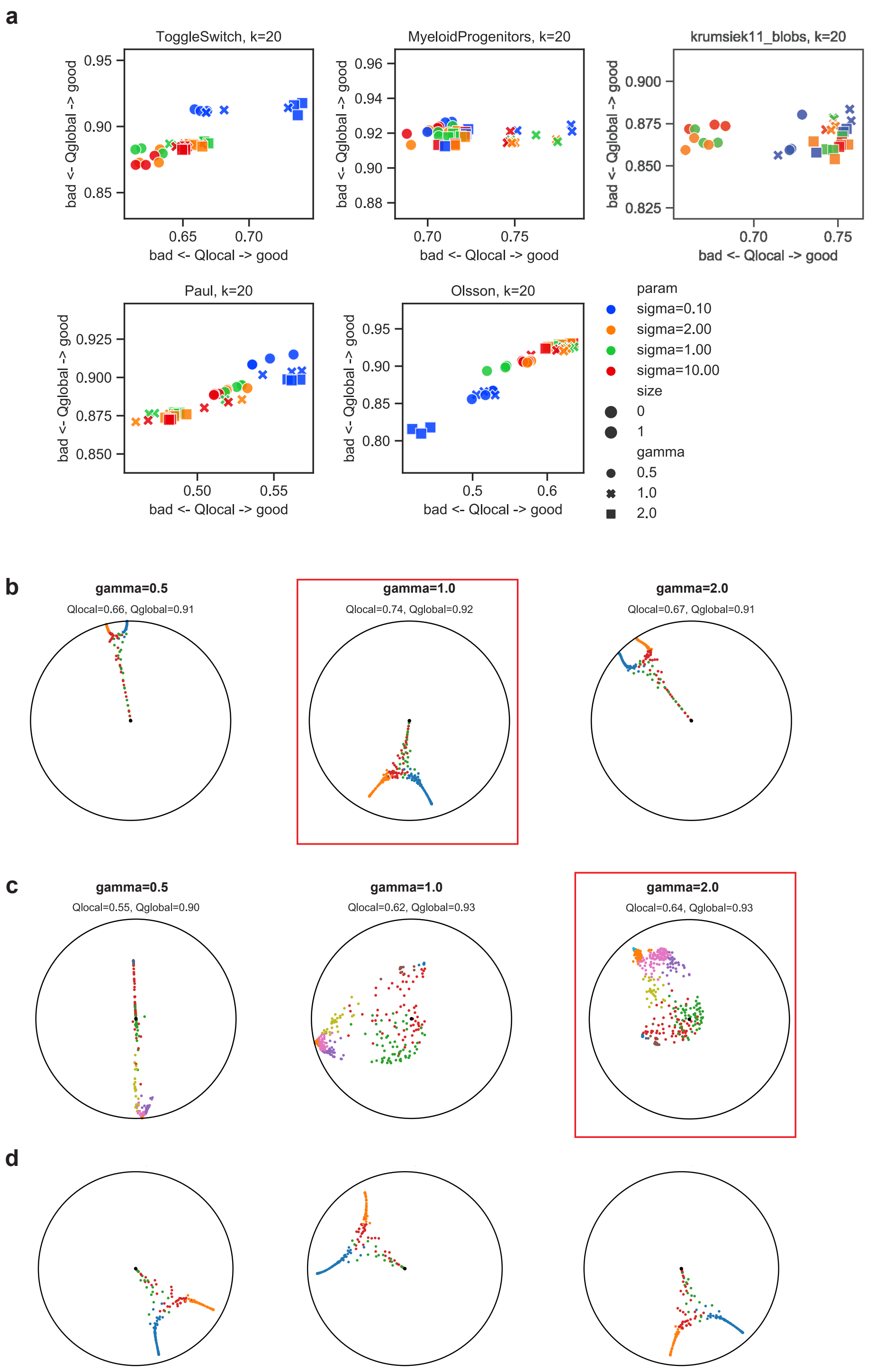

Supplementary Figure 2. Robustness of Poincaré maps to random seed and hyper-parameters choice for a fixed $k = 20$ (a) Scale-independent quality criteria for various hyper-parameters and three random seeds. **(b − c)** Change of visual qualities of the embedding for a fixed σ and varying γ for ToggleSwitch (b) and Olsson (c) datasets. Red frame represents best quality score. **(d)** Comparison of robustness to random seed: σ and γ are fixed.

Supplementary Figure 6 (c) shows how widely used methods such as tSNE distort the pairwise distances, therefore making more difficult to draw conclusions about hierarchies. Similarly, two dimensions of diffusion maps are not enough to represent the branching. UMAP and ForceAtlas2 results overall agree with the Poincaré maps, but don't allow to reason about the subtle hierarchical relations between HSCP-1/2 clusters and MDP. Monocle 2 captures the global branching but fails to depict more fine-grained relations: between erythrocytytes and megakaryocytes or granulocytes and myelocytes.

Mouse myeloid progenitors dataset (MARS-Seq)

As an example of a dataset with multiple intermediate populations, we use a dataset provided by Paul et al. [20]. Myeloid progenitor cells were separated by sorting the c-Kit+ Sca1 lineage from mouse bone marrow and sequenced with MARS-seq. We followed the data preprocessing procedure recipe_zheng17 (Scanpy-recipe [21]), which selects the 1000 most highly-variable genes for 2730 cells. In the original study, the authors identify 19 clusters. We use these labels and canonical hematopoetic cell lineage tree (**Supplementary Figure 7 (a)**) to compare the performance of all methods. We run all methods except Monocle 2 on the 20 top principal components of the preprocessed data. For Monocle 2, we used the Jupyter notebook provided by the authors (the lymphoid cluster was separated as described in the original study).

Supplementary Figure 7 (b) shows the embeddings provided by Poincaré maps. For this dataset, the root is supposed to be at CMP cluster, which is not observed. We chose the root as the medoid (with respect to Poincaré distances) of the MEP and GMP clusters combined. **Supplementary Figure 7 (c)** shows the hierarchy that could be read out from the Poincaré map. We would like to point out that Poincaré maps clearly separate lymphoid cells and dendritic cells as outliers, which agrees with the canonical tree as they are part of lymphoid lineage. None of the other methods (**Supplementary Figure 7 (d)**) were able to capture this fact. Poincaré maps also suggest that some of the clusters (13-15) could be relabeled to better reflect the canonical hierarchy. After the removal of the lymphoid cluster, Monocle 2 captures the main lineage branching between the MEP and GMP lineages, but it does not separate dendritic cells, and destroys the eosonphils cluster. Wolf et al.[9] demonstrated that Monocle 2 results without the removal of the lymphoid cluster only worsen.

Finally, Poincaré maps places the 16Neu cluster downstream of 15Mo in the hierarchy. However, the canonical hierarchy shows neutrophils and monocytes at the same level. As noted by Wolf et al.[9], we suppose that this inconsistency is due to a faulty labeling of the clusters.

Planaria dataset (Drop-seq)

To demonstrate scalability of Poincaré maps to large datasets, we analyzed the entire Planaria dataset of Plass et al. [22]. The dataset comprises 11 individual experiments capturing a total of 21,612 cells with droplet-based single-cell transcriptomics (Drop-seq). To obtain the Poincaré maps we used the pre-processed data provided by the authors: `https://nbviewer.jupyter.org/github/rajewsky-lab/planarian_lineages/blob/master/paga/planaria.ipynb`. The preprocessed dataset comes in the form of 50 principal components, which were used by the authors to apply tSNE, PAGA and ForceAtlas2. (**Supplementary Figure 8**) illustrates that Poincaré maps agree with tSNE and ForceAtlas2 embeddings, significantly outperform PCA and UMAP, and agrees with the PAGA hierarchy annotation (Figure 4 in Plass et al.). Unfortunately we were not able to compute SIMLR for this dataset, because of the computation time.

C. elegans dataset (10X Genomics)

The C. elegans dataset from Packer et al. [23] is the largest dataset used in our experiments. Original dataset contained 84,625 single cells measured with 10x Genomics platform. We used the preprocessed version (batch corrected, 100 PCA components) of the data provided by the authors at `https://github.com/qinzhu/VisCello`. In the original study the authors used UMAP to visualize the data. We loaded the UMAP coordinates provided by the authors to perform fair comparison with Poincaré maps. 37 manually annotated labels of cell types were provided together with the dataset. We randomly down-sampled the dataset to 40,000 cells. The whole dataset represents $>$ 60x oversampling of the 1,341 branches in the C. elegans embryonic lineage, therefore down-sampling should not destroy statistical properties of the dataset. We checked that

sub-sampled data contained all 37 original cell types. **Supplementary Figures 1 , 9, 10** demonstrates, that Poincaré maps significantly outperform all other embedding methods. Unfortunately we were not able to compute SIMLR for this dataset, because of the computation time.

Clustering

Poincaré maps provide embeddings useful beyond visualization. Since Poincaré maps preserve pairwise similarities, their embeddings are suitable for downstream tasks, such as clustering. We compared several clustering approached using Poincaré maps and benchmark embeddings. We also provide Louvain clustering and clustering in the original gene expression space. Since the datasets comprise several continuous trajectories and there is no true separation for progenitor populations of different branches, we used the Adjusted Rand Index (ARI) and Fowlkes-Mallows scores (FMS) to measure cluster quality.

Adjusted Rand Index. The Adjusted Rand Index (ARI) is a function that measures the similarity between two cluster assignments. ARI is bonded between $[-1, 1]$, where negative values correspond to independent labellings, similar clusterings have a positive ARI, and 1.0 is the perfect match score. Lets denote C a ground truth class assignment and K the clustering. Adjusted Rand Index is defined through raw Rand Index (RI):

$$RI = \frac{a+b}{C_2^{n_{samples}}}, \tag{9}$$

where a is the number of pairs of elements that are in the same set in C and in the same set in K, b is the number of pairs of elements that are in different sets in C and in different sets in K, and $C_2^{n_{samples}}$ is the total number of possible pairs in the dataset (without ordering). ARI is after adjusting for random labellings:

$$ARI = \frac{RI - E[RI]}{\max(RI) - E[RI]}, \tag{10}$$

where a is the number of pairs of elements belonging to the same cluster in predicted and true labels, b is the number of pairs of elements belonging to different clusters in predicted and true labels, and $C_2^{n_{samples}}$ is the number of all possible combinations of pairs of elements in the dataset.

Fowlkes-Mallows scores. The Fowlkes-Mallows score FMI is defined as the geometric mean of the pairwise precision and recall:

$$FMI = \frac{TP}{\sqrt{(TP+FP)(TP+FN)}}, \tag{11}$$

where TP is the number of pairs of points that belong to the same cluster in both the true labels and the predicted labels (true positives), FP is the number of pairs of points that belong to the same clusters in the true labels but not in the predicted labels (false positives), and FN is the number of pairs of points that belong in the same clusters in the predicted labels but not in the true labels (false negatives). The FMI ranges from 0 to 1. A high value indicates a good similarity between two clusterings.

More details on these metrics can be found at: https://scikit-learn.org/stable/modules/clustering.html#clustering-evaluation

Supplementary Table 1 shows the clustering results on synthetic datasets. Poincaré maps achieve very similar score to Louvain clustering, and significantly outperform clustering approaches using other embedding methods, except tSNE embedding, which combined with spectral clustering achieves the best scores. However, as we demonstrated before, tSNE does not preserving the hierarchy, and therefore would be less useful for other downstream tasks.

Pseudotime

We demonstrated Poincaré pseudotime performance by comparison with real time and diffusion pseudotime on synthetic datasets. **Supplementary Table 2** demonstrates that Poincaré pseudotime, as well as diffusion pseudotime, achieve high correlation scores with actual time on all synthetic datasets. This is unsurprising since these two measures are related in their nature. The performance of both pseudotime approaches is probably bounded by the construction of kNNG.

Supplementary Note 3: Reconstructing developmental trajectories of the asynchronous process: early blood development in mice (qPCR)

We analyze the single cell qPCR dataset of early blood development in mice [24] using Poincaré maps. We followed the data preprocessing procedure described in Haghverdi et al. [8].

First, we visualized the dataset with a Poincaré map using the labels corresponding to different stages of differentiation [24]: primitive streak (PS), neural plate (NP), head fold (HF), four somite GFP negative (4SG-) and four somite GFP positive (4SG+) (**Supplementary Figure 11 (a)**). We see one cluster standing out. Therefore, we perform spectral clustering with Poincaré distances to break down this cluster for further analysis (**Supplementary Figure 11(b),(c)**). Then, cluster 4 mainly consists of Flk1-Runx1- cells (see **Supplementary Figure 11 (d)**). Moignard et al. [24] refer to this cluster as "mesodermal cells at primitive strike" and suggest that these cells give rise to blood and endothelial cells.

The cell that Haghverdi et al. choose as the root of the differentiation for the diffusion pseudotime analysis belongs to the "mesodermal" cluster in our analysis. We visualize (**Supplementary Figure 12**) the diffusion pseudotime and Poincaré pseudotime with the roots (a) suggested by Haghverdi et al., and (b) the most dissimilar point in the PS cluster in terms of Poincaré distance. Undesirably, the distances from (a) grow orthogonal to the actual developmental stages. It agrees with the conclusion in Haghverdi et al. that such a choice of embedding does not allow to see the asynchronous development. Therefore, cluster 4 may not correspond to cells leading to endothelial and blood cells, but rather to early mesodermal cells, which in their turn lead to some other population (Supplementary Figure 4 in Moignard et al.). We will further refer to cluster 4 as "mesodermal".

As pointed out by Moignard et al., blood development is a highly asynchronous process, which is hard to capture with PCA or diffusion maps. In **Supplementary Figure 13** we further demonstrate how Poincaré maps could be used to reveal the developmental structure in this process. First, we apply the rotation to the Poincaré map to place the root cell defined above to the center of the disk. Then, we apply our lineage detection procedure and demonstrate that inside of each lineage, the order of the developmental stages is on average preserved. However, if we look at all lineages combined, then the populations from PS, NP, HF stages appear to be a homogeneous mixture. Therefore, the angular information in Poincaré maps adds the additional amount of information crucial to understanding asynchronous processes.

Finally, we analyzed the expression profiles of main endothelial and hematopoietic markers for different lineages (**Supplementary Figure 14**). Poincaré maps suggest that cells make an early decision about which branch to become. In particular, we suggest that cells commit to their future branch as early as in the PS stage.

Dataset	ToggleSwitch		Myeloid progenitors		MP with blobs	
name	ARS	FMS	ARS	FMS	ARS	FMS
louvain	0.46	0.59	0.58	0.63	0.89	0.91
spectral Poincaré	0.39	0.54	0.63	0.67	0.89	0.91
agglomerative Poincaré	0.49	0.61	0.59	0.64	0.89	0.91
kmedoids Poincaré	0.38	0.53	0.52	0.59	0.54	0.61
spectral raw	0.18	0.39	0.52	0.58	0.28	0.46
agglomerative raw	0.12	0.42	0.54	0.60	0.46	0.64
kmedoids raw	0.19	0.41	0.55	0.61	0.17	0.36
spectral PCA	0.18	0.39	0.53	0.59	0.29	0.41
agglomerative PCA	0.12	0.42	0.48	0.55	0.43	0.60
kmedoids PCA	0.20	0.42	0.49	0.56	0.65	0.70
spectral tSNE	0.47	0.60	0.59	0.64	0.85	0.88
agglomerative tSNE	0.36	0.51	0.49	0.56	0.89	0.90
kmedoids tSNE	0.43	0.57	0.43	0.51	0.71	0.76
spectral UMAP	0.37	0.52	0.42	0.50	0.89	0.91
agglomerative UMAP	0.37	0.52	0.52	0.58	0.89	0.91
kmedoids UMAP	0.31	0.48	0.58	0.63	0.66	0.71
spectral DiffusionMaps	0.55	0.66	0.52	0.58	0.76	0.81
agglomerative DiffusionMaps	0.04	0.39	0.11	0.31	0.12	0.44
kmedoids DiffusionMaps	0.06	0.36	0.42	0.50	0.33	0.50
spectral ForceAtlas2	0.00	0.53	-0.00	0.30	0.00	0.39
agglomerative ForceAtlas2	0.48	0.61	0.56	0.61	0.89	0.91
kmedoids ForceAtlas2	0.42	0.56	0.55	0.61	0.53	0.60

Supplementary Table 1. Comparison of various clustering approaches on the synthetic datasets. Higher values are better.

Dataset	dpt	pmpt	dpt-pmpt
ToggleSwitch: branch1	0.99	0.99	0.99
ToggleSwitch: branch2	0.98	0.98	0.99
ToggleSwitch: avg	0.99	0.98	0.99
MyeloidProgenitors: erythrocyt	0.89	0.94	0.91
MyeloidProgenitors: megakaryoc	0.94	0.95	0.93
MyeloidProgenitors: monocyte	0.93	0.89	0.98
MyeloidProgenitors: neutrophil	0.91	0.91	0.99
MyeloidProgenitors: avg	0.92	0.92	0.95
MyeloidProgenitors with blobs: Ery	0.89	0.94	0.90
MyeloidProgenitors with blobs: Mk	0.94	0.96	0.93
MyeloidProgenitors with blobs: Mo	0.93	0.89	0.97
MyeloidProgenitors with blobs: Neu	0.91	0.91	0.99
MyeloidProgenitors with blobs: avg	0.92	0.92	0.95

Supplementary Table 2. Comparison of diffusion pseudotime (dpt) and Poincaré pseudotime (pmpt) against real time on synthetic datasets using Pearson correlation coefficient between. The last column corresponds to the correlation coefficient between diffusion pseudotime and Poincaré pseudotime. Higher values are better.

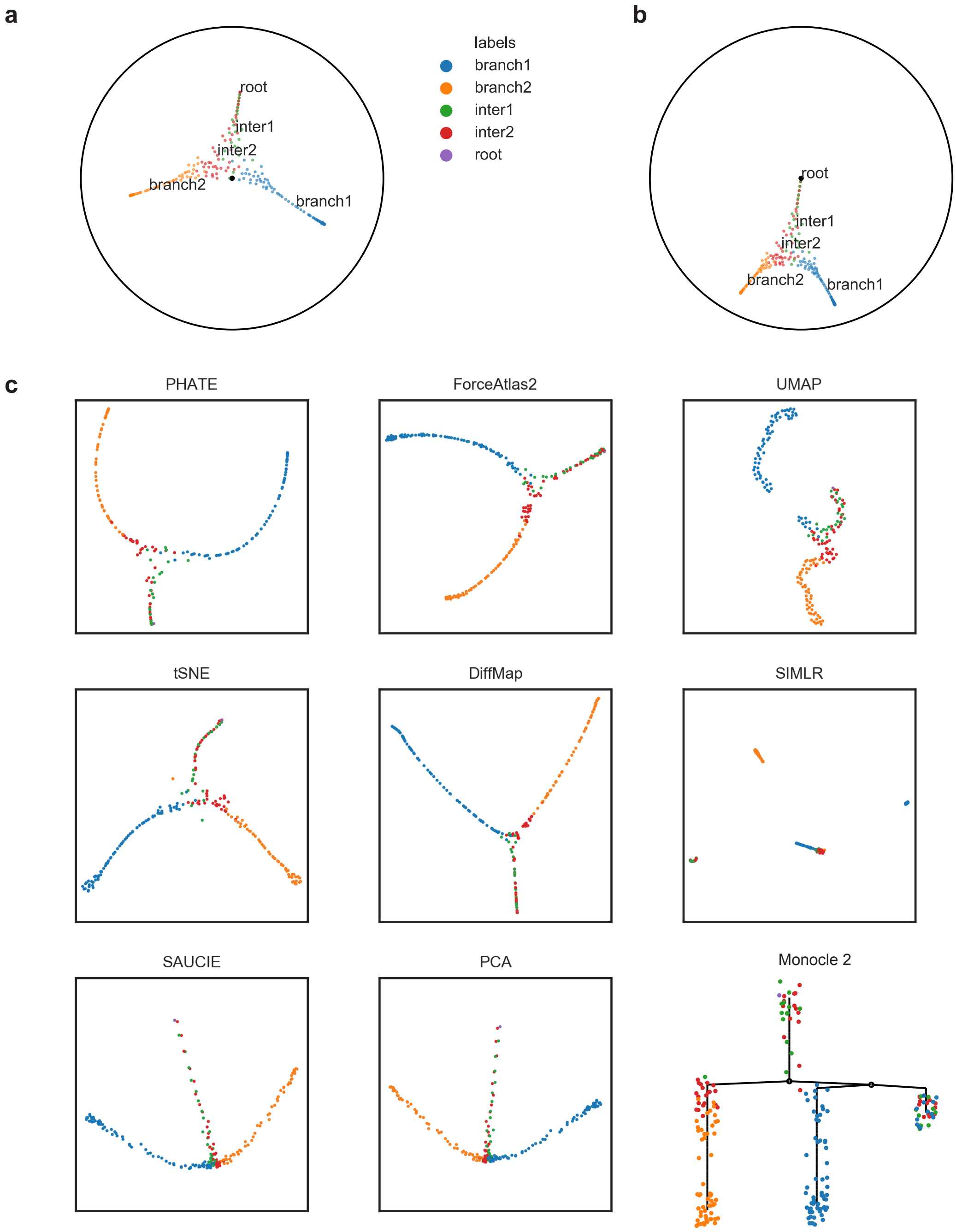

Supplementary Figure 3. Comparison of various embeddings for the simple toggle switch model. There are two distinct branches. We additionally labeled intermediate states from the simulations. **(a)** Raw Poincaré map. **(b)** Rotation of the Poincaré map with respect to the known root. **(c)** Benchmark methods.

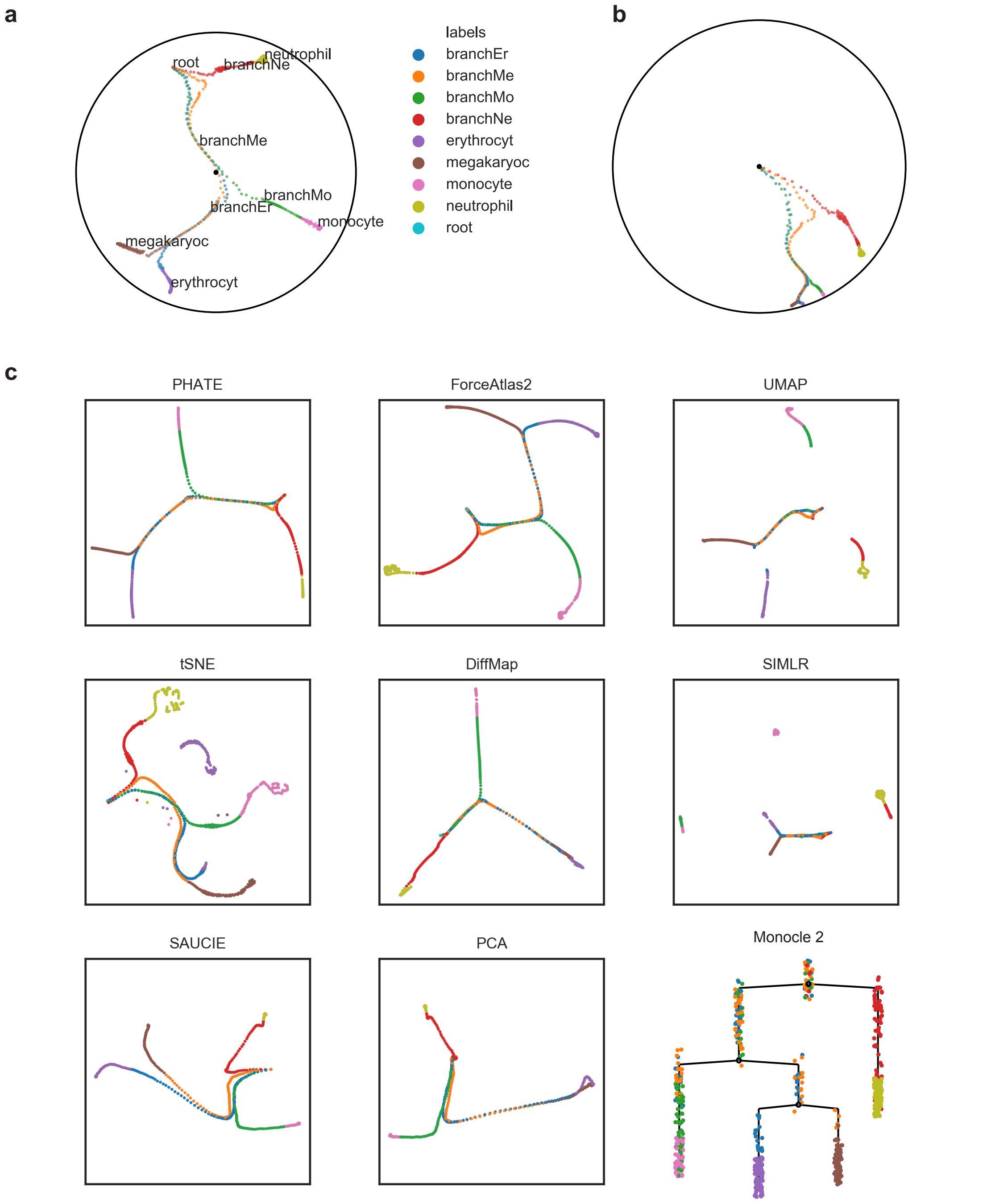

Supplementary Figure 4. Comparison of various embeddings for a synthetic model of myeloid progenitors differentiation. There are four distinct branches. We additionally labeled intermediate states from the simulations. **(a)** Raw Poincaré map. **(b)** Rotation of the Poincaré map with respect to the known root. **(c)** Benchmark methods.

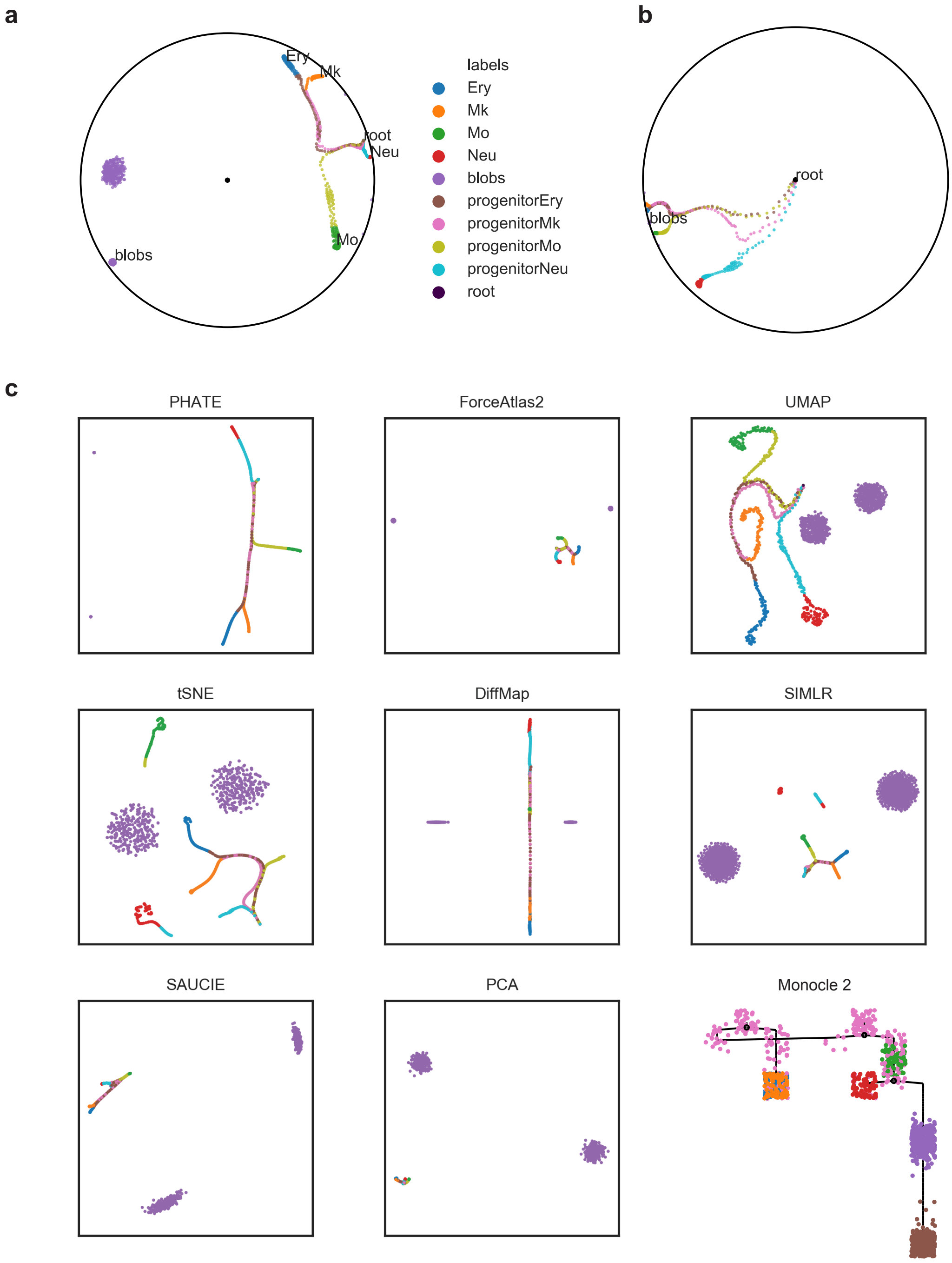

Supplementary Figure 5. Comparison of various embeddings for a synthetic model of myeloid progenitors differentiation (4 distinct branches) with two additional Gaussian clusters. We additionally labeled intermediate states from the simulations. **(a)** Raw Poincaré map. **(b)** Rotation of the Poincaré map with respect to the known root. **(c)** Benchmark methods.

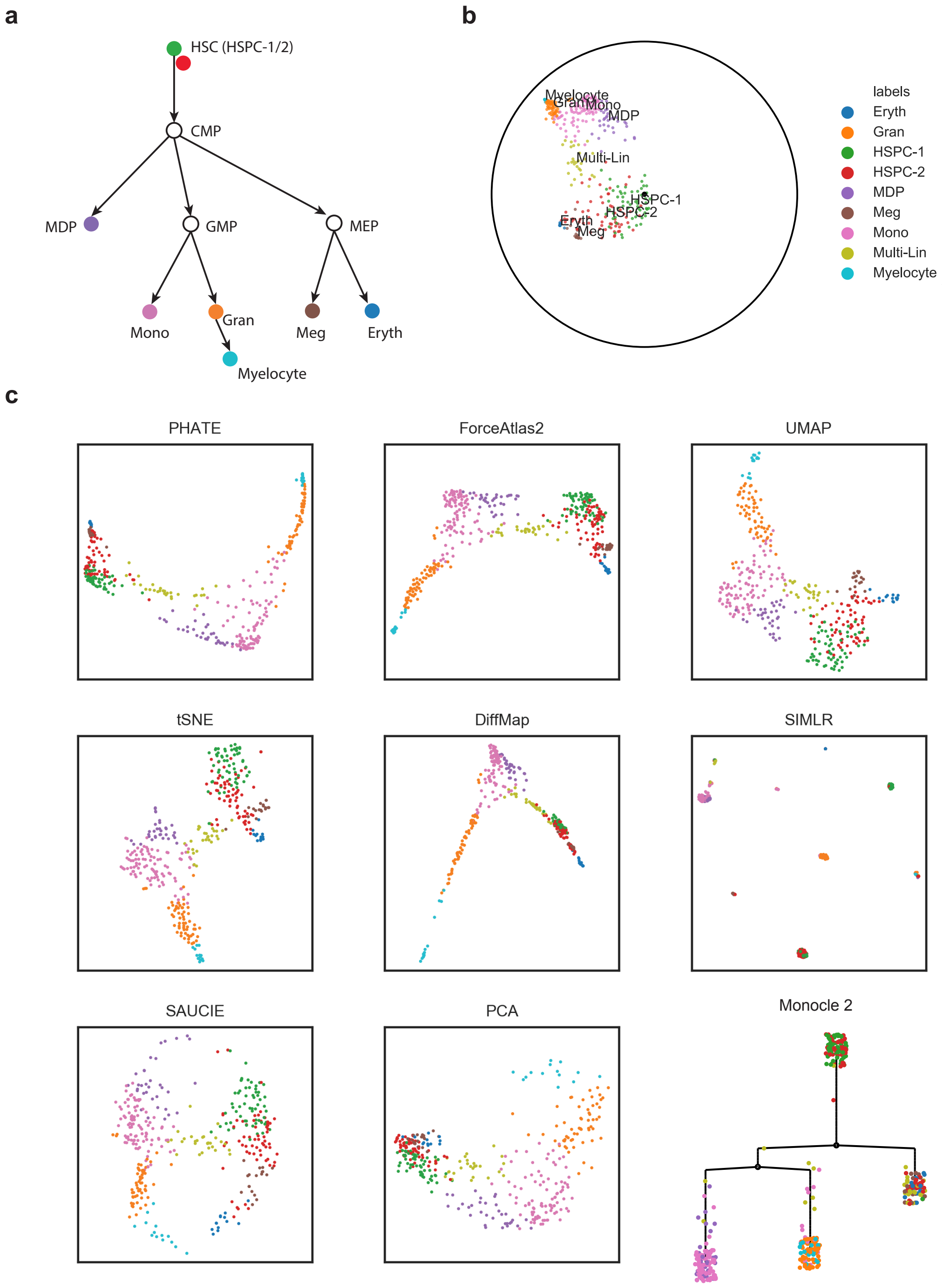

Supplementary Figure 6. Comparison of various embeddings for the scRNAseq dataset of mouse myelopoesis (Olsson et al.). **(a)** Canonical hematopoetic cell lineage tree. Colored circles correspond to the population colors from the dataset. **(b)** Poincaré map rotated with respect to the root. **(c)** Benchmark methods.

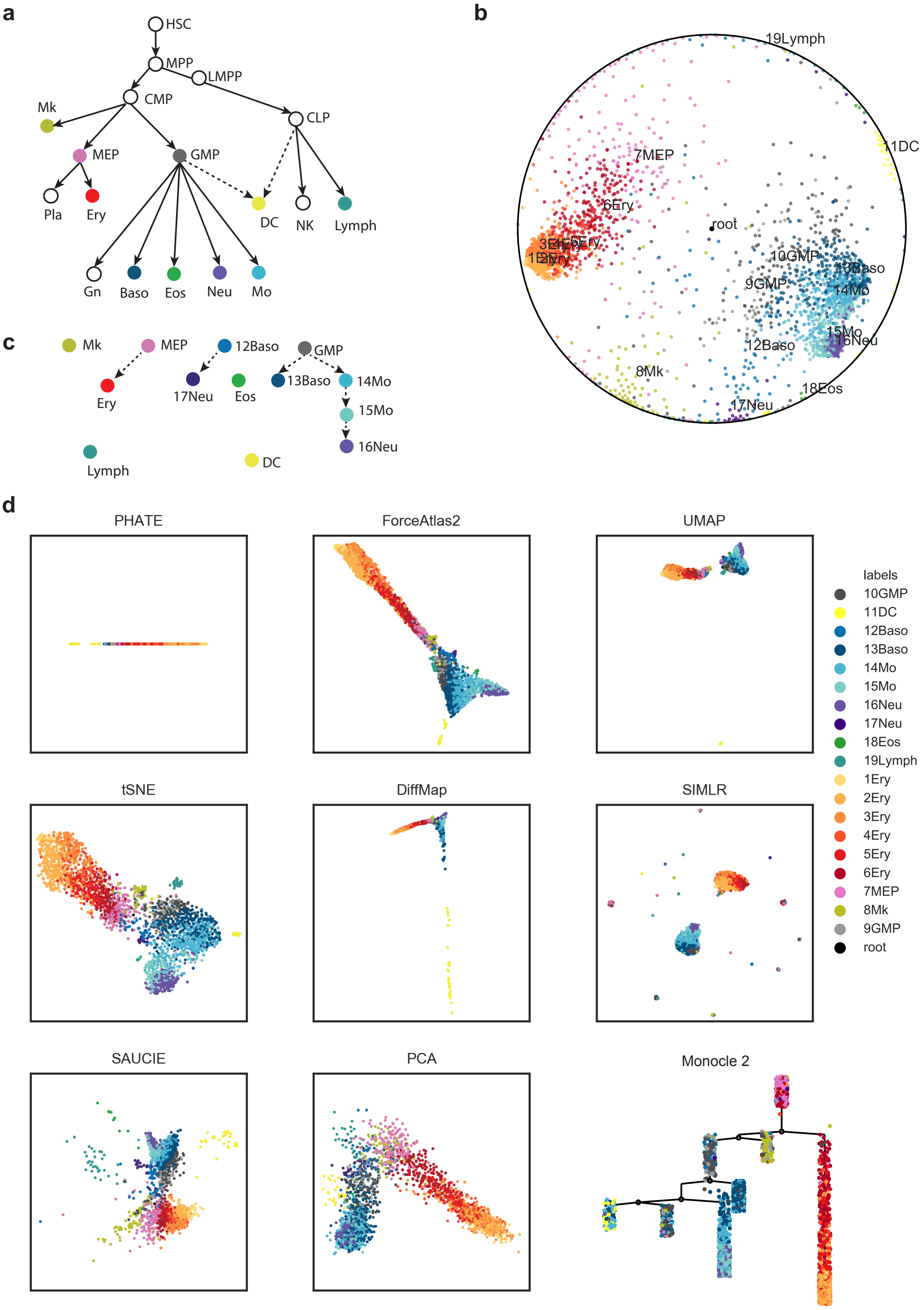

Supplementary Figure 7. Comparison of various embeddings for the mouse myeloid progenitors MARS-seq dataset (Paul et al.). (a) Canonical hematopoetic cell lineage tree. Colored nodes correspond to the population colors from the dataset. White nodes correspond to intermediate annotated states. **(b)** Rotated Poincaré map with respect to the root (medoids of MEP and GMP cluster). **(c)** Hierarchical relationships suggested by the Poincaré map. **(d)** Benchmark methods. To reproduce the Monocle 2 tree, the lymphoid cluster was removed.

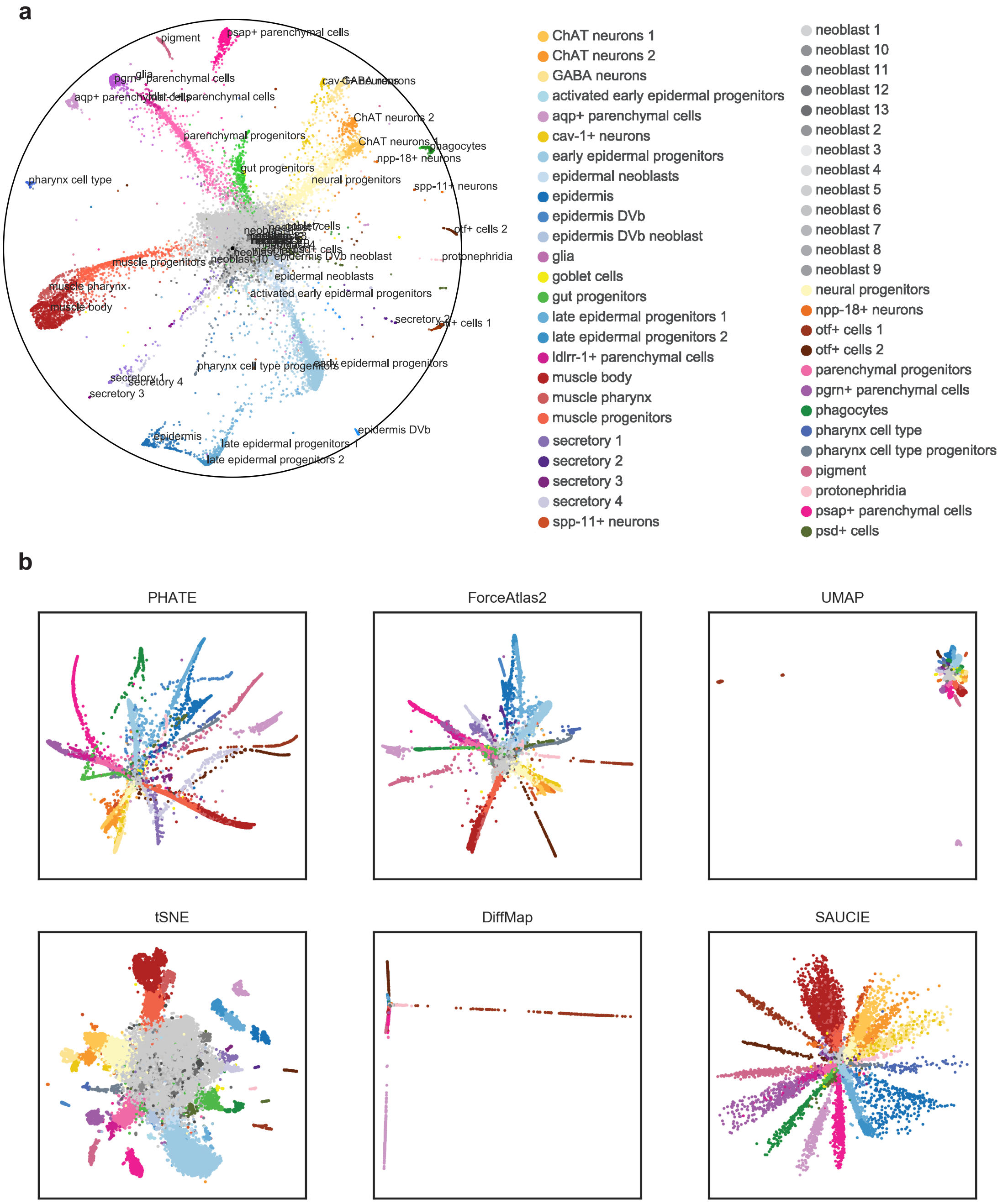

Supplementary Figure 8. Comparison of various embeddings for the planaria Drop-seq dataset (Plass et al.). (a) Poincaré map rotated with respect to the root (medoids of neoblast 1 cluster). **(b)** Benchmark methods.

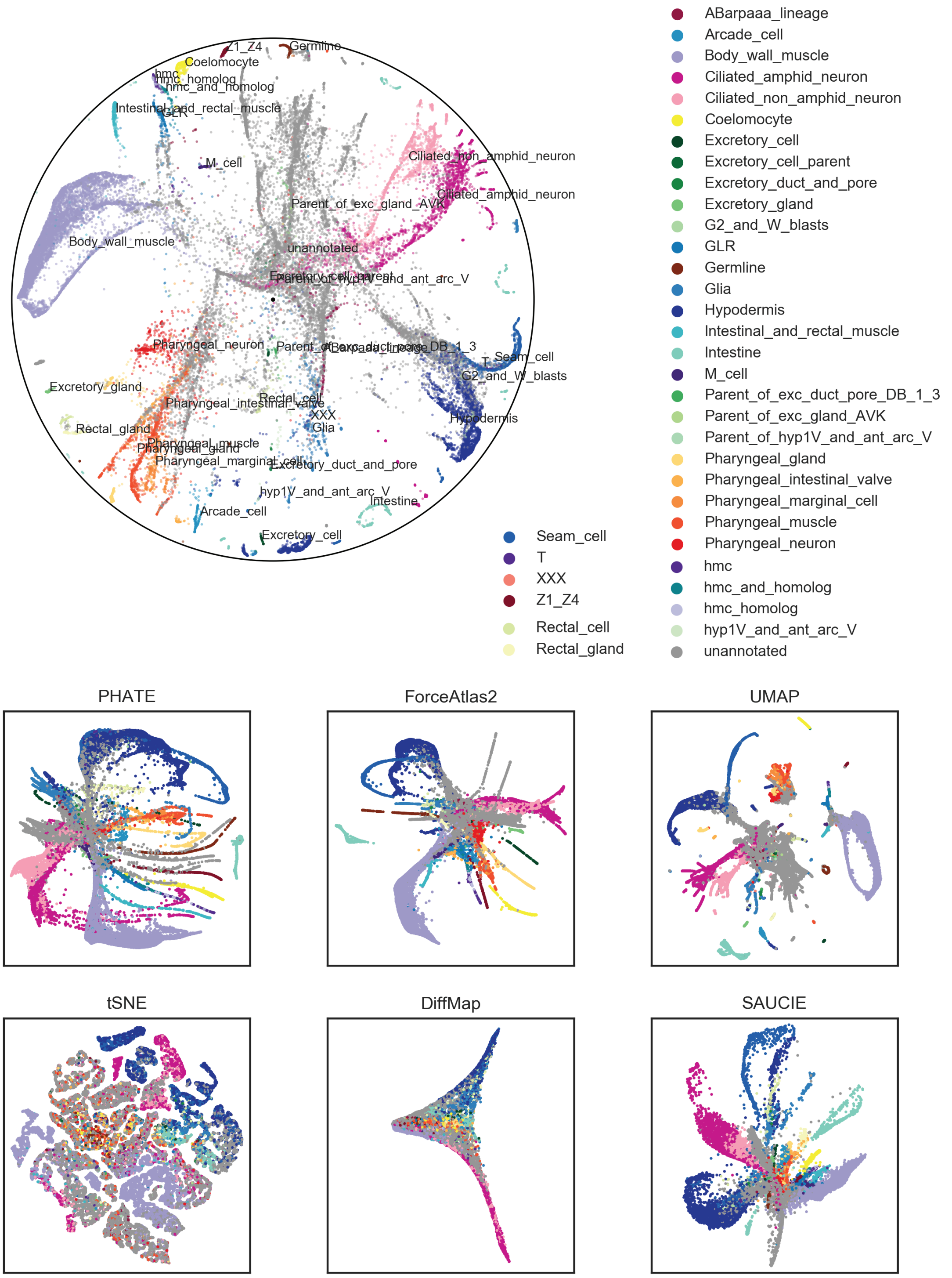

Supplementary Figure 9. Comparison of various embeddings for the C. elegans 10X Genomics dataset (Packer et al.).

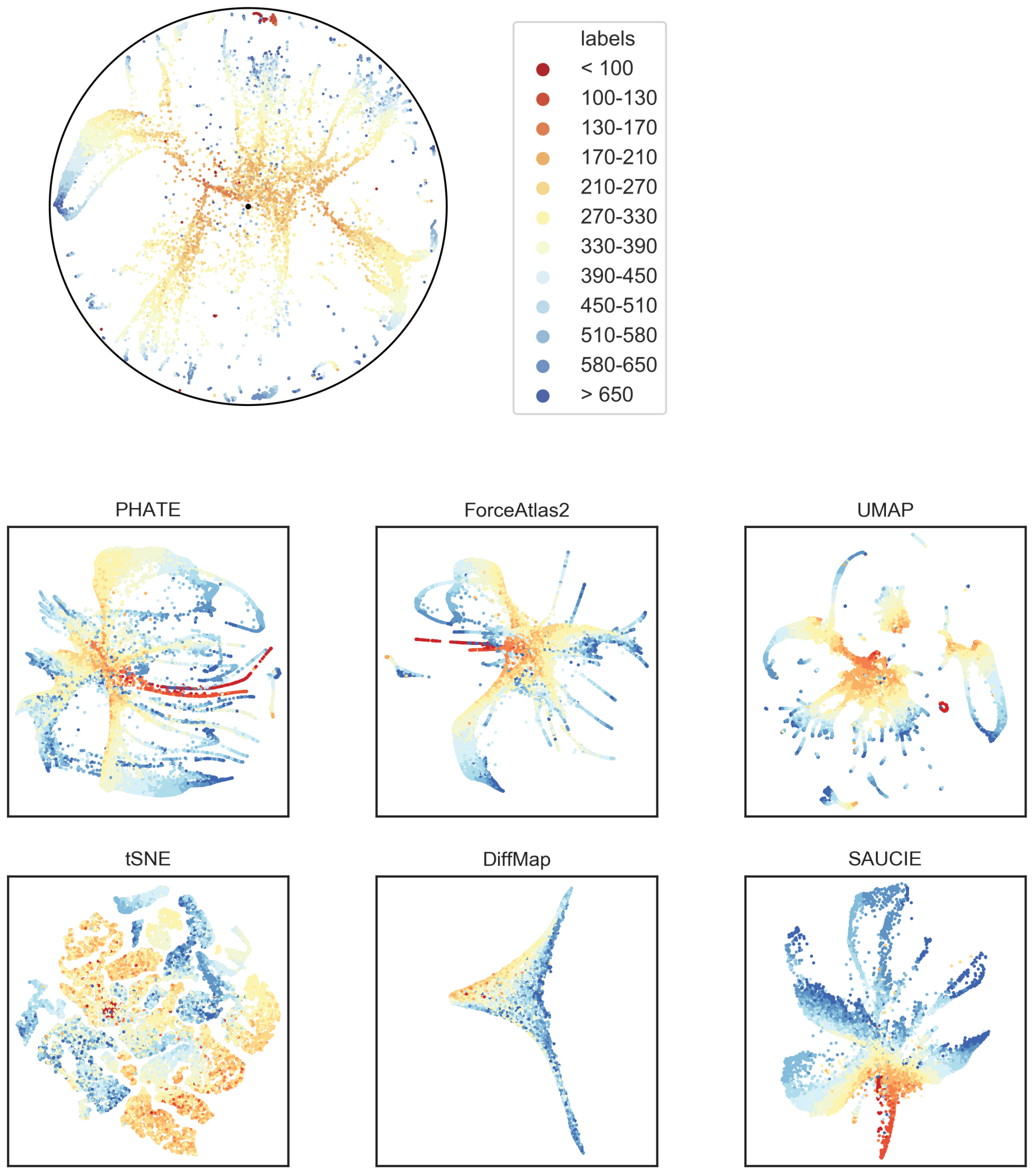

Supplementary Figure 10. Comparison of the age of the embryo for various embeddings for the C. elegans 10X Genomics dataset (Packer et al.).

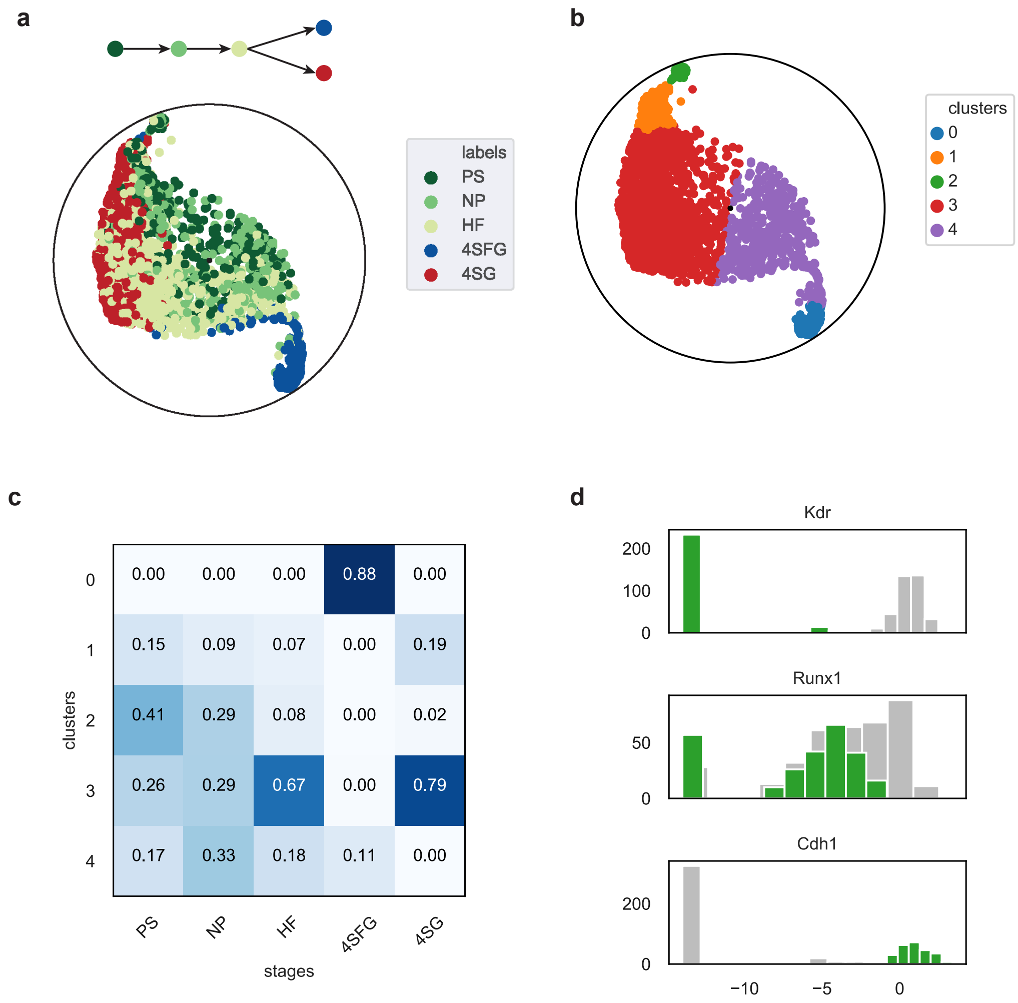

Supplementary Figure 11. **(a)** Poincaré map of the Moignard dataset. **(b)** Spectral clustering with Poincaré distances. **(c)** Analysis of stage-composition of the defined clusters. Clusters 1 and 3 most likely represent development of blood cells. Clusters 0 and 4 potentially correspond to endothelial development. Cluster 2 corresponds to the cluster named "mesodermal cells at primitive strike" in the original paper. **(d)** Comparison of the median expression of markers at PS stage for cluster 2 against the rest of PS cells. Cluster 4 consists mostly of Flk1-Runx1-.

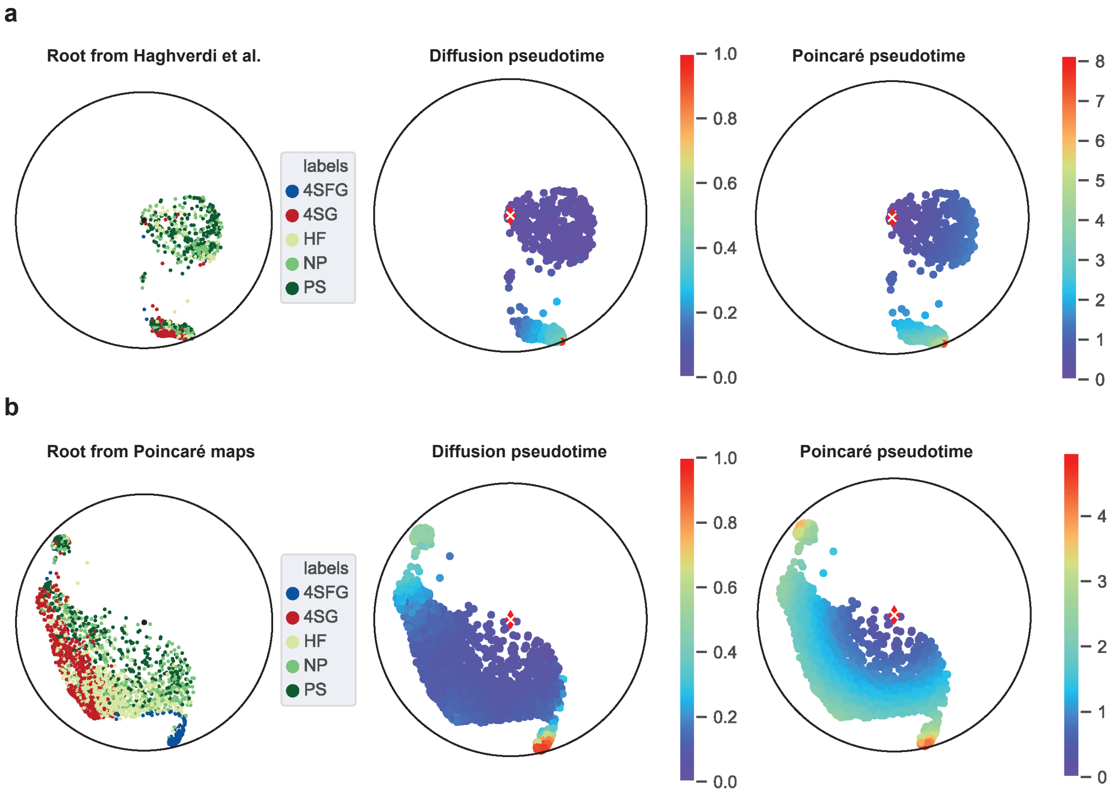

Supplementary Figure 12. Rotation of the Poincaré map, and corresponding pseudotimes with root chosen according to **(a)** Haghverdi et al. **(b)** proposed new root.

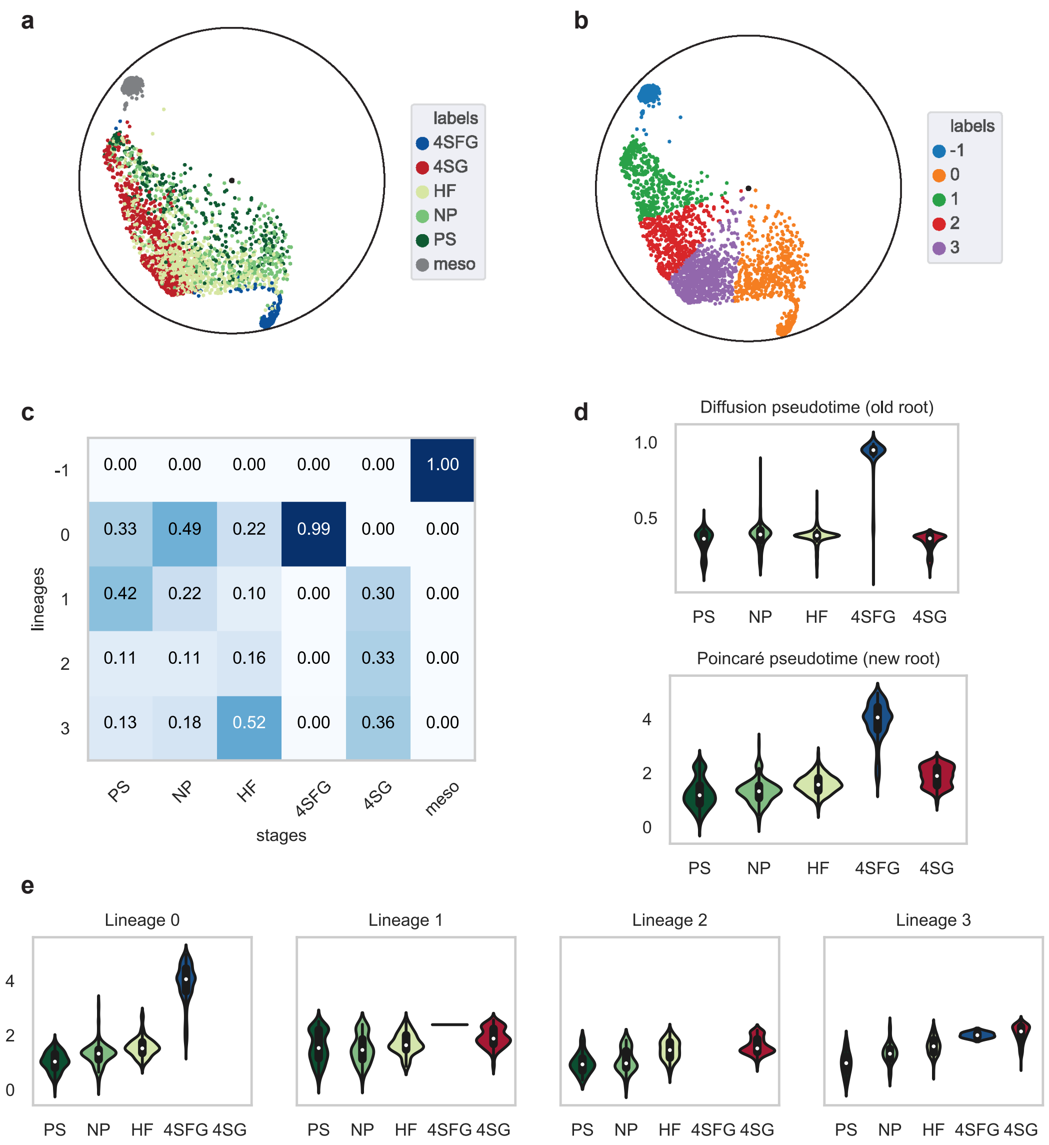

Supplementary Figure 13. Analysis of stage ordering in different lineages. **(a)** Poincaré map with developmental stages. **(b)** Detected lineages with clustering by angle in the Poincaré disk. **(c)** Lineage composition per stage. **(d)** Average diffusion (from Haghverdi et al.) and Poincaré pseudotime per stage for the whole dataset. **(e)** Average Poincaré pseudotime per stage for in the individual lineage.

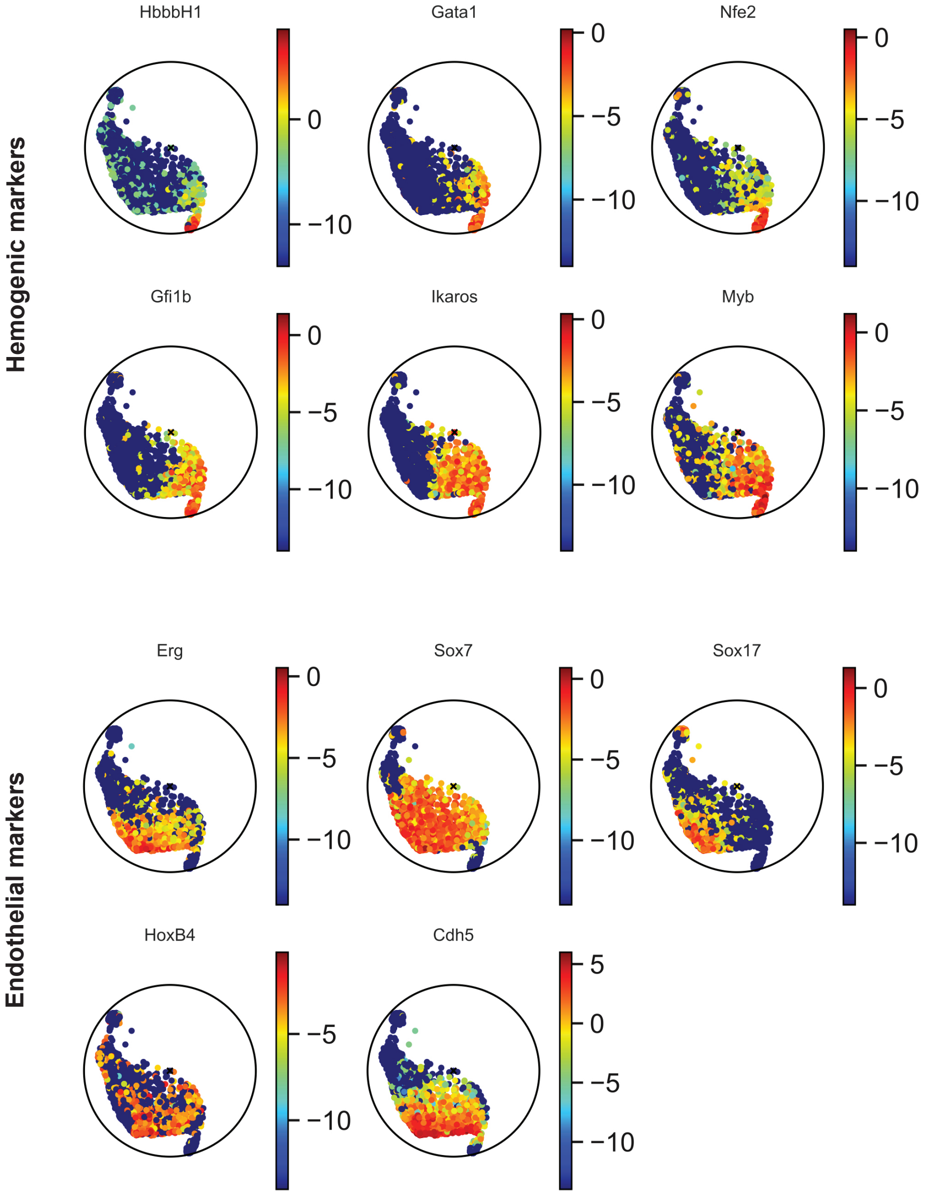

Supplementary Figure 14. Expression of main endothelial and hemogenic markers visualized on the Poincaré disk.

Supplementary References

[1] Gromov, M. *Metric structures for Riemannian and non-Riemannian spaces* (Springer Science & Business Media, 2007).

[2] Nickel, M. & Kiela, D. Poincaré embeddings for learning hierarchical representations. In Guyon, I. *et al.* (eds.) *Advances in Neural Information Processing Systems 30*, 6338–6347 (Curran Associates, Inc., 2017). URL http://papers.nips.cc/paper/7213-poincare-embeddings-for-learning-hierarchical-representations.pdf.

[3] De Sa, C., Gu, A., Ré, C. & Sala, F. Representation tradeoffs for hyperbolic embeddings. *Proceedings of machine learning research* **80**, 4460 (2018).

[4] Nickel, M. & Kiela, D. Learning continuous hierarchies in the lorentz model of hyperbolic geometry. In *Proceedings of the Thirty-fifth International Conference on Machine Learning* (2018).

[5] Bonnabel, S. Stochastic gradient descent on Riemannian manifolds. *IEEE Trans. Automat. Contr.* **58**, 2217–2229 (2013). URL http://dx.doi.org/10.1109/TAC.2013.2254619.

[6] Maaten, L. v. d. & Hinton, G. Visualizing data using t-sne. *Journal of machine learning research* **9**, 2579–2605 (2008).

[7] McInnes, L. & Healy, J. Umap: Uniform manifold approximation and projection for dimension reduction. (2018). Preprint at https://arxiv.org/abs/1802.03426.

[8] Haghverdi, L., Buettner, M., Wolf, F. A., Buettner, F. & Theis, F. J. Diffusion pseudotime robustly reconstructs lineage branching. *Nature methods* **13**, 845 (2016).

[9] Wolf, F. A. *et al.* Paga: graph abstraction reconciles clustering with trajectory inference through a topology preserving map of single cells. *Genome biology* **20**, 59 (2019).

[10] Jacomy, M., Venturini, T., Heymann, S. & Bastian, M. Forceatlas2, a continuous graph layout algorithm for handy network visualization designed for the gephi software. *PloS one* **9**, e98679 (2014).

[11] Qiu, X. *et al.* Reversed graph embedding resolves complex single-cell trajectories. *Nature methods* **14**, 979 (2017).

[12] Lee, J. A. & Verleysen, M. Scale-independent quality criteria for dimensionality reduction. *Pattern Recognition Letters* **31**, 2248–2257 (2010).

[13] Wolf, F. A., Angerer, P. & Theis, F. J. Scanpy: large-scale single-cell gene expression data analysis. *Genome biology* **19**, 15 (2018).

[14] Wittmann, D. M. *et al.* Transforming boolean models to continuous models: methodology and application to t-cell receptor signaling. *BMC systems biology* **3**, 98 (2009).

[15] Gardner, T. S., Cantor, C. R. & Collins, J. J. Construction of a genetic toggle switch in escherichia coli. *Nature* **403**, 339 (2000).

[16] Haghverdi, L., Buettner, F. & Theis, F. J. Diffusion maps for high-dimensional single-cell analysis of differentiation data. *Bioinformatics* **31**, 2989–2998 (2015).

[17] Krumsiek, J., Marr, C., Schroeder, T. & Theis, F. J. Hierarchical differentiation of myeloid progenitors is encoded in the transcription factor network. *PloS one* **6**, e22649 (2011).

[18] Olsson, A. *et al.* Single-cell analysis of mixed-lineage states leading to a binary cell fate choice. *Nature* **537**, 698 (2016).

[19] Murphy, K. & Weaver, C. *Janeway's immunobiology* (Garland Science, 2016).

[20] Paul, F. *et al.* Transcriptional heterogeneity and lineage commitment in myeloid progenitors. *Cell* **163**, 1663–1677 (2015).

[21] Zheng, G. X. *et al.* Massively parallel digital transcriptional profiling of single cells. *Nature communications* **8**, 14049 (2017).

[22] Plass, M. *et al.* Cell type atlas and lineage tree of a whole complex animal by single-cell transcriptomics. *Science* **360**, eaaq1723 (2018).

[23] Packer, J. S. *et al.* A lineage-resolved molecular atlas of c. elegans embryogenesis at single-cell resolution. *Science* **365**, eaax1971 (2019).

[24] Moignard, V. *et al.* Decoding the regulatory network of early blood development from single-cell gene expression measurements. *Nature biotechnology* **33**, 269 (2015).
